# Femtosecond laser-assisted in situ keratomileusis with topography-guided or asphericity-adjusted derived data: a comparative contralateral eye study

**DOI:** 10.1186/s12886-022-02407-w

**Published:** 2022-04-25

**Authors:** Ermano M. Alves, Adriana F. Lyra, Manuela Tenório, Natália Mesquita, Carolina Bacelar, Afra Montenegro, Lucas Alves, Márcio Alves

**Affiliations:** 1 Oftalmax, Rua Benfica, 411, Madalena, Recife, PE 50720-001 Brazil; 2 Hospital Santa Luzia, Estrada do Encanamento, 909, Casa Forte, Recife, PE Brazil; 3 FAV, R da Soledade 170, Recife, PE Brazil; 4 FPS-IMIP, Av Mal. Mascarenhas de Morais, Recife, PE 4861 Brazil

**Keywords:** Femtosecond LASIK, femtoLASIK, Topography-guided LASIK, Asphericity-guided LASIK, T-CAT, Custom-Q, Contoura

## Abstract

**Background:**

Wavefront-optimized laser-assisted in situ keratomileusis (LASIK) ablation is the most commonly performed procedure in refractive surgery, but new technologies have become available. Our goal was to compare topography-guided (Contoura) and asphericity-guided (Custom-Q) customized ablation treatments for the correction of myopia with or without astigmatism.

**Methods:**

This prospective, randomized, double-blind, contralateral eye study included 60 eyes of 30 patients with myopia or myopic astigmatism requiring femtosecond LASIK (FemtoLASIK) treatment. For each patient, one eye was randomized to undergo Contoura treatment, and the other underwent Custom-Q abaltion. Uncorrected distance visual acuity (UDVA), corrected distance visual acuity (CDVA), manifest refractive spherical equivalent (MRSE), sphere (SPH), cylinder (CYL), 6.0-mm total corneal aberration root mean square (RMS), coma (COMA), trefoil (TREF), and spherical aberration (SA) were measured and analysed after a 1-year follow-up.

**Results:**

The UDVA was − 0.08 ± 0.06 logMAR in Contoura eyes and − 0.08 ± 0.05 logMAR in Custom-Q eyes (*p* = 0.309) after 12 months. Twenty-five eyes (83%) in the Contoura group and twenty-six eyes (87%) in the Custom-Q group had a UDVA of 20/16 at the end of 12 months, and 100% of eyes in both groups reached a UDVA of 20/25 or better. Ninety and 100% of eyes in the Contoura and Custom-Q groups, respectively, achieved a residual CYL ≤0.50 D (*p* = 0.237). No statistically significant difference was observed between the surgical techniques in the preoperative to 1-year postoperative changes for any of the parameters evaluated (MRSE, CYL, RMS, DEF, COMA, TREF, and SA).

**Conclusions:**

The Contoura and Custom-Q techniques yielded excellent visual and refractive results, but the evidence did not reveal any clear differences between these two methods after 1 year of follow-up.

**Trial registration:**

ReBEC - Registro Brasileiro de Ensaios Clínicos [Internet]: Rio de Janeiro (RJ): Instituto de Informação Científica e Tecnológica em Saúde (Brazil); 2010 -. Identifier RBR-8rs5kt Myopia and Astigmatism Topography-guided Refractive Surgery by Contoura Method Versus Customized by Asphericity in Contralateral Eyes: A prospective Double blind Randomized Study. Available from https://ensaiosclinicos.gov.br/rg/RBR-8rs5kt

Date of registration: 02/03/2020 ^(dd/mm/yyyy)^.

CAAE:96778718.9.0000.5192.

Issuing authority: Plataforma Brasil.

CEP:2.979.279.

Issuing authority: HUOC.

## Background

The prevalence of myopia is increasing; approximately 50% of the world population is estimated to present with this condition by 2050 [1]. At the same time, the evolution of refractive surgery towards higher levels of safety and satisfaction has led to the development of different treatment techniques and ablation profiles with excimer lasers [2, 3]. Customized treatments of refractive errors have been available for many years, involving wavefront-, topography-, asphericity-, or ablation-guided optimizations that have achieved outcomes superior to those observed with conventional ablation [4–8].

Typically, the human cornea is aspherical and prolate (i.e., it is more curved at the centre and flatter at the periphery). However, conventional refractive surgeries for myopia correction induce changes in corneal asphericity, primarily by turning the prolate shape of the corneal surface into an oblate shape (i.e., flatter at the centre and more curved at the periphery). These changes can increase ocular aberrations, specifically spherical aberrations, which in excessive amounts may result in postoperative poor visual acuity in low-light conditions [8].

Physiological corneal asphericity varies significantly across individuals and can be measured by using a concept called the Q factor [9]; a Q factor of − 1 to 0 indicates a prolate corneal surface, while a Q factor > 0 indicates and oblate surface, and a spherical shape is represented by a Q factor of 0 [9]. Normal corneas usually have a minimal Q value ranging from − 0.23 to − 0.30 and a positive spherical aberration (SA) of approximately + 0.27 μm [10]. The Q-factor customized corneal ablation aims to correct refractive errors by modifying as little as possible the preoperative Q and SA.

Approximately 90% of optical aberrations of the human eye are derived from corneal surface irregularities [11]. Over the years, the application of topography-guided excimer laser ablation in irregular corneas, decentred or small optic zones after refractive surgery and even in ectatic corneas has been optimized with excellent results [12–15]. Several studies have suggested using this technique in normal corneas, and the results have been shown to be superior to those obtained with currently used ablation techniques [4–6, 8].

In 2013, the U.S. Food and Drug Administration (FDA) approved the use of WaveLight® topography-guided customized ablation treatment (T-CAT) [16] in the United States for treating myopia in regular corneas with or without astigmatism. This type of treatment aims to correct small irregularities in normal corneas that are responsible for high-order optical aberrations (HOAs), which, at least in principle, influence the final clinical refraction. Following correction of these small changes by the topography-guided laser, the final refraction to be treated must also experience small changes. This approach would thus lead to better visual acuity and fewer optical aberrations with respect to other techniques [10].

Potential modifications in topography-based refraction for achieving a visual acuity greater than 20/20 and better visual quality, however, require further investigation, and to date, there is no consensus on the criteria for modifying the final treatment and whether this approach is superior to other ablation profiles already widely used by refractive surgeons.

In this study, we aimed to compare two methods of corneal ablation performed using femtosecond laser-assisted in situ keratomileusis (LASIK) in myopic eyes with or without astigmatism in virgin eyes. For each patient, one eye was treated with T-CAT, and the contralateral eye was treated with Custom-Q. Both methods required the use of WaveLight software.

## Methods

### Study design

This prospective, randomized, double-blind, comparative study was conducted at the OFTALMAX Eye Clinic and Surgery in Recife, Pernambuco, Brazil. As this is a pilot study, sample size was not calculated according to the conventional power analysis method.

This study was a per protocol analysis of 51 patients selected between August and December 2018 at the Santa Luzia Foundation in Recife, an ophthalmological care facility affiliated with the Unified Health Service in Brazil. The study was approved by the ethics committee of the Hospital Complex HUOC/PROCAPE after informed consent was and was conducted following the guidelines of the Declaration of Helsinki. The study included patients aged 20 to 35 years of both sexes with myopia with or without astigmatism, with a stable refractive error ranging between − 0.50 and − 8.00 spherical dioptres (D) and astigmatism between 0.00 and − 3.00 cylindrical dioptres (CD) for a minimum of 1 year, a maximum spherical equivalent of 8.00 D, and a corrected visual acuity of 0.1 logMAR or better. The exclusion criteria were any clinical condition that could modify the surgical outcome, such as anisometropia with a refractive error of more than 1.00 D in spherical or 0.75 D in astigmatism, clinical signs of dry eye, cataracts, corneal scarring, or neovascularization within 1.0 mm of the intended ablation zone; epithelial basement membrane dystrophy; history of recurrent corneal erosion; pachymetry below 500 μm; suspicion of subclinical or established keratoconus; macular or retinal disease; diagnosis of glaucoma or ocular hypertension; current use of systemic corticosteroids or immunosuppressive therapy; collagen diseases; vascular diseases; diabetes mellitus types I and II; pregnancy and breastfeeding.

### Preoperative examinations

Preoperative examinations included measurement of visual acuity using a digital projector (Apramed CB 300) without distance correction and then with cycloplegic refraction after 30 min using 1% cyclopentolate hydrochloride (Allergan); measurement of intraocular pressure using a Goldmann tonometer; anterior segment biomicroscopy; retinal mapping by indirect ophthalmoscopy; complete anterior segment analysis including 6.0-mm total corneal aberration root mean square (RMS), coma (COMA), trefoil (TREF), and spherical aberration (SA) using dual Scheimpflug-based corneal system tomography (Galilei Ziemer Ophthalmic Systems AG, Port, Switzerland); measurement of asphericity and corneal HOAs using Wavelight Topolyzer Vario software (Alcon Laboratories, Inc., Fort Worth, Texas) and acquisition of a minimum of eight, good-quality, reproducible images. The images were digitally transferred to an EX500 (Alcon) Excimer Laser workstation and used to plan the ablation profile. Patients were asked to discontinue wearing contact lenses, if applicable, 1 week before the screening (soft contact lenses) or 4 weeks before screening (rigid gas-permeable contact lenses).

### Surgeries

The surgeries were performed on two consecutive days and involved the entire team of investigators and two Alcon WaveLight consultants. All preoperative examination outcomes were assessed, and images were acquired on the day of the surgery via Topolyzer Vario (WaveLight) software. All patients were scheduled for simultaneous FemtoLASIK in both eyes. Visual correction was performed according to the cycloplegic refraction with T-CAT using the commercially called Contoura method in group 1 and Custom-Q in group 2 (contralateral eye); final goal was to achieve emmetropia in all patients.

Randomization was ensured using a spreadsheet created in Excel. Consequently, following the order of the patients admitted to the block, even-numbered patients were administered Contoura treatment in the right eye and Custom-Q in the left eye, while the odd-numbered patients were treated with Contoura in their left eye and Custom-Q in the right eye.

Contoura surgical planning was performed following the guidelines updated by Alcon technical engineers according to the following steps:

1. Acquisition of a minimum of eight good-quality topographic images using Topolyzer Vario software.
2. Comparison between the pupillary and mid-peripheral areas using the Compare Images Display and identification of four or more images with a refractive error of less than 0.75 D in the treatment area demonstrating a good definition of the limbus and pupillary margin.
3. Transfer of images via a USB stick to the EX500 (Alcon) Excimer Laser workstation. Images with a value of 3 were considered high-quality (i.e., good quality for eventual cyclotorsion control) using the Quality Dynamic Link Library.
4. Exclusion of lower-quality images with a refractive error ≥ 0.75 D in the treatment area until a minimum of four images were obtained with a mean average deviation (MAD) of the axis of astigmatism of 0.5.
5. Import of cycloplegic refraction data into Contoura planning software.
6. Zeroing of astigmatism and spherical degree to visualize the ablation pattern of the HOAs;
7. Determination of the amount of ablated tissue in the central and peripheral regions of the cornea. The difference was not permitted to exceed 3 μm; if this occurred, the spherical correction was altered until this limit was achieved.
8. Observation of the difference between the topographic cylinder measured using Topolyzer Vario software and the manifest refractive cylinder, with final treatment corrected according to the following parameters:
  a- When the refractive cylinder was smaller than the topographic cylinder, the average between the two measurements was obtained, and treatment was performed with the final mean value while maintaining the topographic axis.
  b- When the refractive cylinder was larger than the topographic cylinder, treatment was performed with the axis and the total cylinder of the topographic cylinder.
9. Computation of the spherical degree requiring correction after adjusting the spherical equivalent of the change in astigmatism power. This value could vary according to the WaveLight nomogram.

The Custom-Q treatment was performed targeting the previous asphericity (Q) of each patient, which was measured by the Topolyzer Vario and automatically exported to the pre-surgical planning; furthermore, the HOAs of the cornea were left uncorrected. Both treatments were centred on the corneal vertex.

Safety was ensured by measuring the percentage of altered tissue (PTA) using the corneal thickness at its thinnest region when calculating the ablation [17, 18]. All surgeries were performed by an experienced surgeon (EMA) at the OFTALMAX operating room using a WaveLight® EX500 (Alcon) Excimer Laser according to data provided by Topolyzer Vario software (WaveLight). A corneal flap was created using a femtosecond Z8 laser (Ziemer Ophthalmic Systems, Switzerland). The flap thickness was 110 μm, the flap diameter was 9.0–9.5 mm, and the optical zone was 6.5 mm in all treatments. The surgeon did not have access to the type of treatment applied to each eye.

### Postoperative follow-up

In the immediate postoperative period, topical treatment was initiated with a combination of moxifloxacin and dexamethasone (Vigadexa, Alcon) four times a day for 1 week, and lubricant eye drops containing sodium hyaluronate and carboxymethylcellulose (Optive, Allergan) were provided by Oftalmax. The patients were examined by medical researchers who did not participate in the randomization at day 1, week 1, month 1, 3 months and 1 year after the procedure.

During the first three postoperative follow-up visits, possible complications and ocular surface integrity were assessed by anterior segment biomicroscopy, uncorrected distance visual acuity (UDVA), and fundoscopy. The last two visits included the evaluation of the patients’ visual acuity with cycloplegic refraction, retinal mapping, applanation tonometry, and total anterior segment analysis including RMS and HOAs performed by dual Scheimpflug-based corneal system tomography.

### Statistical methodology

Quantitative data are expressed as the mean, standard deviation, median, minimum, and maximum. Qualitative or categorical variables are described as absolute (n) and relative (%) frequencies and were compared using the chi-square test or Fisher’s exact test, in the case of 2 × 2 tables, with expected frequencies < 5.

The Kolmogorov–Smirnov test was used to assess whether the quantitative data had a normal distribution. Student’s t-test was used to compare the two surgical techniques for data considered normally distributed, and when normality could not be assumed, the nonparametric Mann–Whitney U test was used. The paired t-test was used to assess differences between pre- and postoperative parameters under the assumption of normality, and the nonparametric Wilcoxon test was used in case the data were not normally distributed.

Regarding the assessment of uncorrected distance visual acuity (UDVA) measured at day 1 (D1), week 1 (W1), 3 months (3 M), and 1 year (1Y), a mixed ANOVA model was used for comparing surgical techniques and measurements over time and assessing the interaction effect between the factors “surgery” and “time”. Regarding the statistical significance of variation over time, the Bonferroni method for multiple comparisons was used for comparisons with the baseline value at day 1 of the surgery.

All analyses were performed using Minitab 18.0 software. *p* values < 0.05 were considered statistically significant.

## Results

### Demographic data

Among the 51 patients initially selected for the study, 13 were excluded. After PTA analysis, five patients did not appear to be good candidates for flap creation and underwent photorefractive keratectomy; one presented with a flap complication (the vertical cut for the flap margin was not performed), and a second flap was required after 1 h in the same eye; four did not have reliable images produced according to the T-CAT criteria; one mistakenly underwent bilateral T-CAT ablation; one was purposely under corrected in both eyes to avoid exceeding the 40% PTA limit; and one developed severe dry eye keratitis in both eyes, preventing reliable data collection. The final sample included a total of 38 participants, but among them, 8 did not participate in the 1-year final evaluation.

A total of 30 patients and 60 surgeries were evaluated (30 in the right eye and 30 in the left eye). Patients were between 19 and 33 years old, with a mean age of 26.3 years and standard deviation of 4.3 years. Just over half of the patients were female (56.7%) (Table 1). The sequence of surgeries in the right and left eyes was not balanced, as the Contoura technique was mostly used on the right eye, while the Custom-Q technique was mostly used on the left eye (73.3%) (Table 2).

**Table 1:** Demographic data

Demographic data	Total
No. of patients	30
No. of eyes	60
Age, years	
N	30
mean (sd)	26.3 (4.3)
median	26
min–max	19–33
Sex, N (%)	
women	17 (56.7%)
men	13 (43.3%)

*sd* standard deviation, *min* minimum observed value, *max* maximum observed value

**Table 2:** Operated eye versus surgical technique used

	Contoura (*n* = 30)	Custom-Q (*n* = 30)	Total (*n* = 60)
right eye, n (%)	22 (73.3%)	8 (26.7%)	30 (50%)
left eye, n (%)	8 (26.7%)	22 (73.3%)	30 (50%)
total, n (%)	30 (100%)	30 (100%)	60 (100%)

### Comparison of surgical techniques in the pre- and intraoperative periods

No statistically significant difference was found when comparing the Contoura and Custom-Q groups in the parameters measured in the preoperative period (CDVA, spherical equivalent, sphere, cylinder, Sim K, pachymetry), nor in the PTA measured during surgery (Table 3).

**Table 3:** Comparison of techniques with regard to the parameters evaluated in the pre- and intraoperative periods

	Contoura (*n* = 30)	Custom-Q (*n* = 30)	*p*-value
CDVA (logMAR)			
mean ± sd	−0.06 ± 0.06	− 0.06 ± 0.05	
median	−0.1	− 0.1	0.679^1^
min–max	−0.1–0.1	−0.1–0	
Spher. Equiv. (D)			
mean ± sd	−3.39 ± 1.65	−3.47 ± 1.69	
median	−2.88	−2.94	0.813^1^
min–max	− 7.75 – − 1.63	− 7.88 – − 1.00	
Sphere (D)			
mean ± sd	− 2.88 ± 1.53	− 3,03 ± 1.60	
median	− 2.50	−2.50	0.678^1^
min–max	− 7.25 – − 1.25	−7.00 – − 1.00	
Cylinder (D)			
mean ± sd	− 1.02 ± 0.83	− 0.88 ± 0.71	
median	− 0.88	−0.75	0.527^1^
min–max	−3.25–0.00	− 2.50–0.00	
Sim K (D)			
mean ± sd	43.92 ± 1.49	43.99 ± 1.48	
median	43.89	43.95	0.850^2^
min–max	40.00–48.02	40.36–47.70	
Pachy (D)			
mean ± sd	543.7 ± 28.30	544.57 ± 29.20	
median	542.00	542.00	0.872^2^
min–max	493.00–600.00	494.00–607.00	
PTA (%)			
mean ± sd	30.97 ± 3.13	30.12 ± 3.12	
median	30.10	30.11	0.322^1^
min–max	25.20–39.00	23.80–37.20	

*sd* standard deviation, *min* minimum observed value, *max* maximum observed value, *D* dioptre

^1^Mann–Whitney test

^2^Student t-test

### Evolution of parameters between the preoperative evaluation and 1-year follow-up

Both surgical techniques achieved significant increases in the value of parameters, such as the root mean square (RMS), defocus (DEF) and coma (COMA). The SA parameter also presented significant variation between the pre- and postoperative assessments, with a median of − 0.18 D in the preoperative assessment and − 0.27 D and − 0.29 D in the postoperative assessment in the Contoura and Custom-Q groups, respectively.

Only the trefoil (TREF) parameter presented with no significant variation in value in the 1-year follow-up relative to the preoperative period, with *p* = 0.198 (Contoura) and *p* = 0.972 (Custom-Q) (Table 4).

**Table 4:** Comparison of pre- and postoperative values according to the type of surgery performed

	Contoura (*n* = 30)			Custom-Q (*n* = 30)		
	pre	post	*p*-value	pre	post	*p*-value
RMS (μm)						
mean ± sd	1.07 ± 0.45	1.67 ± 0.53		0.99 ± 0.46	1.76 ± 0.59	
median	0.93	1.63	< 0.001^1^	0.92	1.61	< 0.001^1^
min–max	0.49–2.43	0.77–3.14		0.33–2.19	0.74–3.25	
DEF (D)						
mean ± sd	0.28 ± 0.23	1.07 ± 0.40		0.24 ± 0.23	1.09 ± 0.43	
median	0.32	1.06	< 0.001^2^	0.29	1.06	< 0.001^2^
min–max	−0.15–0.66	0.31–2.16		−0.29–0.69	0.34–2.11	
COMA (D)						
mean ± sd	0.18 ± 0.09	0.26 ± 0.15		0.17 ± 0.08	0.29 ± 0.14	
median	0.19	0.24	0.017^2^	0.17	0.25	< 0.001^2^
min–max	0.02–0.47	0.05–0,81		0.04–0.31	0.07–0.64	
TREF (D)						
mean ± sd	0.13 ± 0.08	0.13 ± 0.08		0.13 ± 0.08	0.15 ± 0.07	
median	0.13	0.11	0.198^1^	0.12	0.13	0.972^1^
min–max	0.03–0.41	0.01–0.31		0.02–0.36	0.03–0.37	
SA (D)						
mean ± sd	−0.17 ± 0.04	−0.28 ± 0.09		− 0.17 ± 0.05	−0.32 ± 0.12	
median	−0.18	− 0.27	< 0.001^2^	− 0.18	−0.29	< 0.001^2^
min–max	−0.24 – − 0.05	−0.51 – − 0.06		−0.28 – − 0.03	−0.65 – − 0.13	

*sd* standard deviation, *min* minimum observed value, *max* maximum observed value, *D* dioptre

^1^t-paired test

^2^Wilcoxon test

### Comparison of surgical techniques between pre- and postoperative assessments (1-year follow-up)

No statistically significant differences were observed between the surgical techniques in terms of the preoperative to 1-year postoperative changes for the five parameters evaluated at the 1-year follow-up (RMS, DEF, COMA, TREF, and SA) (Table 5).

**Table 5:** Comparison of the preoperative to 1-year postoperative changes in parameters between the types of surgery

	Difference between pre- and postoperative value (1 year)		
	Contoura	Custom-Q	*p*-value
RMS (μm)			
mean ± sd	0.59 ± 0.68	0.77 ± 0.67	
median	0.58	0,82	0.311^1^
min–max	−0.81–2.32	− 0.61–1.96	
DEF (D)			
mean ± sd	0.79 ± 0.34	0.85 ± 0.40	
median	0.71	0.78	0.712^2^
min–max	0.31–1.67	0.26–2.16	
COMA (D)			
mean ± sd	0.07 ± 0.16	0.12 ± 0.14	
median	0.07	0.10	0.221^1^
min–max	−0.22–0.56	−0.11–0.44	
TREF (D)			
mean ± sd	−0.001 ± 0.10	0.02 ± 0.08	
median	−0.01	0.01	0.402^1^
min–max	−0.19–0.22	−0.24–0.19	
SA (D)			
mean ± sd	−0.10 ± 0.10	−0.14 ± 0.13	
median	−0.09	− 0.10	0.294^2^
min–max	−0.43–0.06	−0.50–0.00	

*sd* standard deviation, *min* minimum observed value, *max* maximum observed value, *D* dioptre

^1^Student t-test

^2^Mann–Whitney test

### Assessment of UDVA

**Fig. 1:**
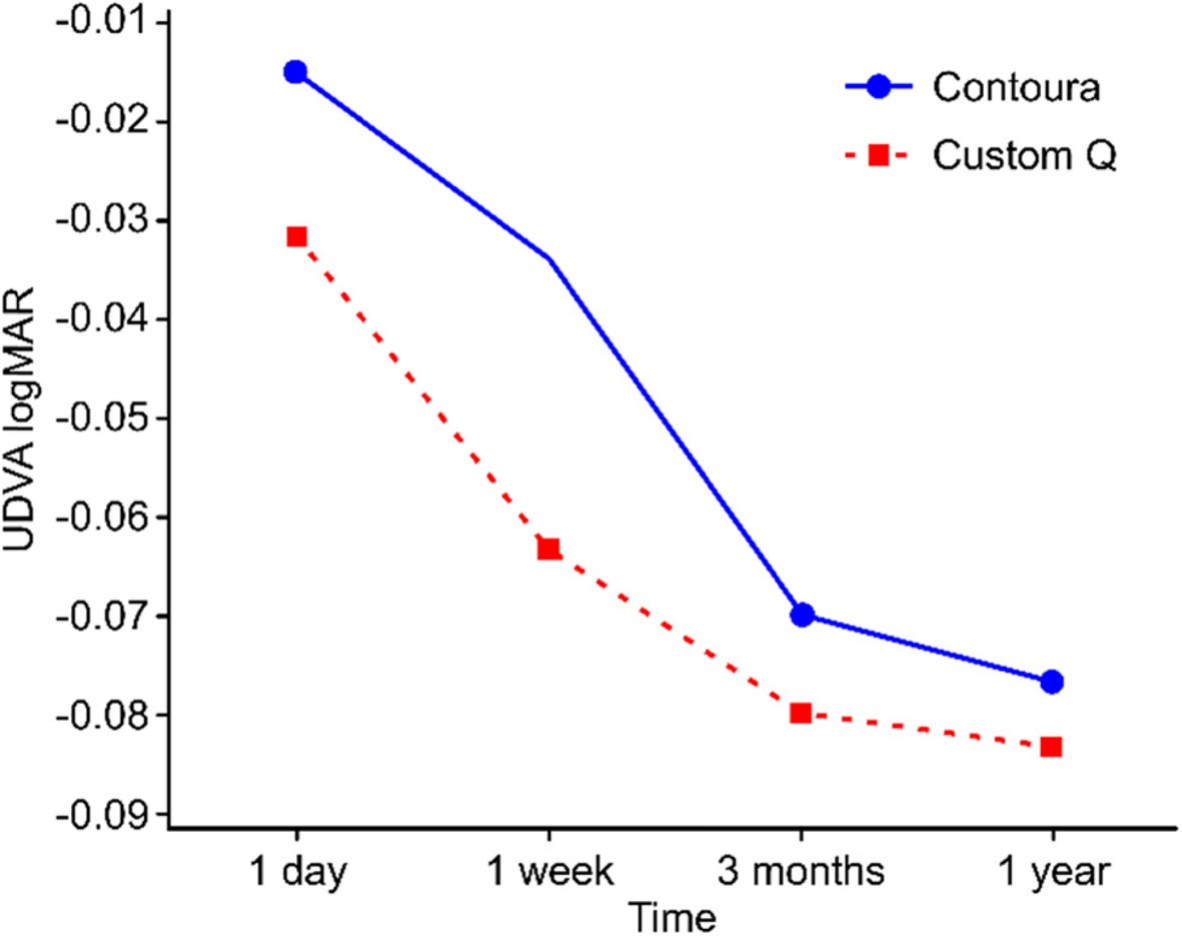
Uncorrected distance visual acuity assessment (UDVA) (mean). Embedded text: Interaction Graph for UDVA, UDVA (mean logMAR), Surgery Contoura/Custom-Q, Day 1/Week 1/3 months/1 year, Assessment

No interaction was identified between surgical techniques and assessments (*p* = 0.727), indicating that the curves representing the surgeries showed a similar behaviour over time (Fig. 1).

Thus, the analysis of the UDVA data showed that regardless of the assessment performed, there were no significant differences between the surgical techniques (*p* = 0.309), but a significant improvement between the assessments at W1, 3 M, and 1Y relative to D1 (*p* < 0.001) was identified for both groups (Table 6).

**Table 6:** Uncorrected distance visual acuity (logMAR)

UDVA	Contoura	Custom-Q
Day 1 (baseline)		
mean ± sd	−0.01 ± 0.12	−0.03 ± 0.11
median	− 0.05	−0.10
min–max	−0.10–0.40	−0.12–0.40
Week 1		
mean ± sd	−0.03 ± 0.07	−0.06 ± 0.06
median	0.00	−0.10
min–max	−0.10–0.18	−0.10–0.10
3 months		
mean ± sd	−0.07 ± 0.07	−0.08 ± 0.05
median	− 0.10	−0.10
min–max	−0.30–0.10	−0.10–0.10
1 year		
mean ± sd	−0.08 ± 0.06	−0.08 ± 0.05
median	− 0.10	−0.10
min–max	−0.10–0.10	−0.10–0.10

ANOVA mixed model:

comparison of surgical techniques: *p* = 0.309

comparison of assessments: *p* < 0.001 (multiple comparisons with baseline)

interaction of factors: *p* = 0.727

### Analysis of data from standardized tables: statistical comparison of the Contoura and custom-Q surgical techniques

#### Efficacy

**Fig. 2:**
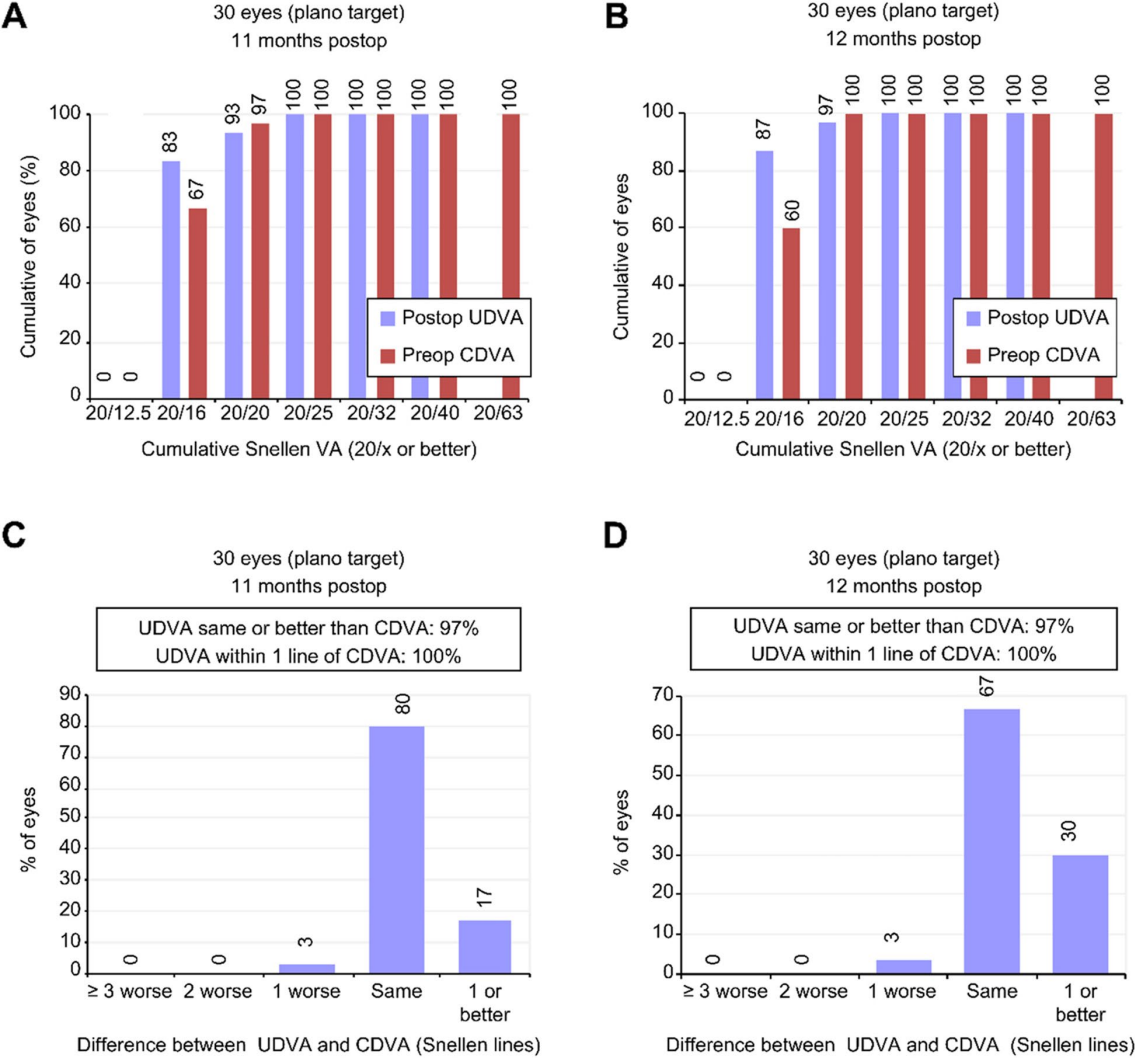
Refractive and visual outcomes, **A** and **C** = Contoura, **B** and **D** = Custom-Q (UDVA = uncorrected distance visual acuity; CDVA = corrected distance visual acuity; VA = visual acuity)

Twenty-five eyes (83%) in the Contoura group and twenty-six eyes (87%) in the Custom-Q group had a UDVA of 20/16 at the end of 12 months, and 100% in both groups reached a UDVA of 20/25 or better. There was no difference between the groups in terms of the UDVA (20/16 versus 20/20 and 20/25; *p* = 1.000, Fisher’s exact test) or preoperative CDVA (20/16 versus 20/20 and 20/25; *p* = 0.592, chi-square test) (Fig. 2A and B).

#### Postoperative UDVA vs. preoperative CDVA

Only one eye in each group (3%) experienced loss of one line of sight between preoperative CDVA and postoperative UDVA. There was gain of one line of sight or more in five eyes (17%) in the Contoura group and nine eyes (30%) in the Custom-Q group, but this difference was not statistically significant (*p* = 0.243, chi-square test) (Fig. 2C and D).

#### Safety

**Fig. 3:**
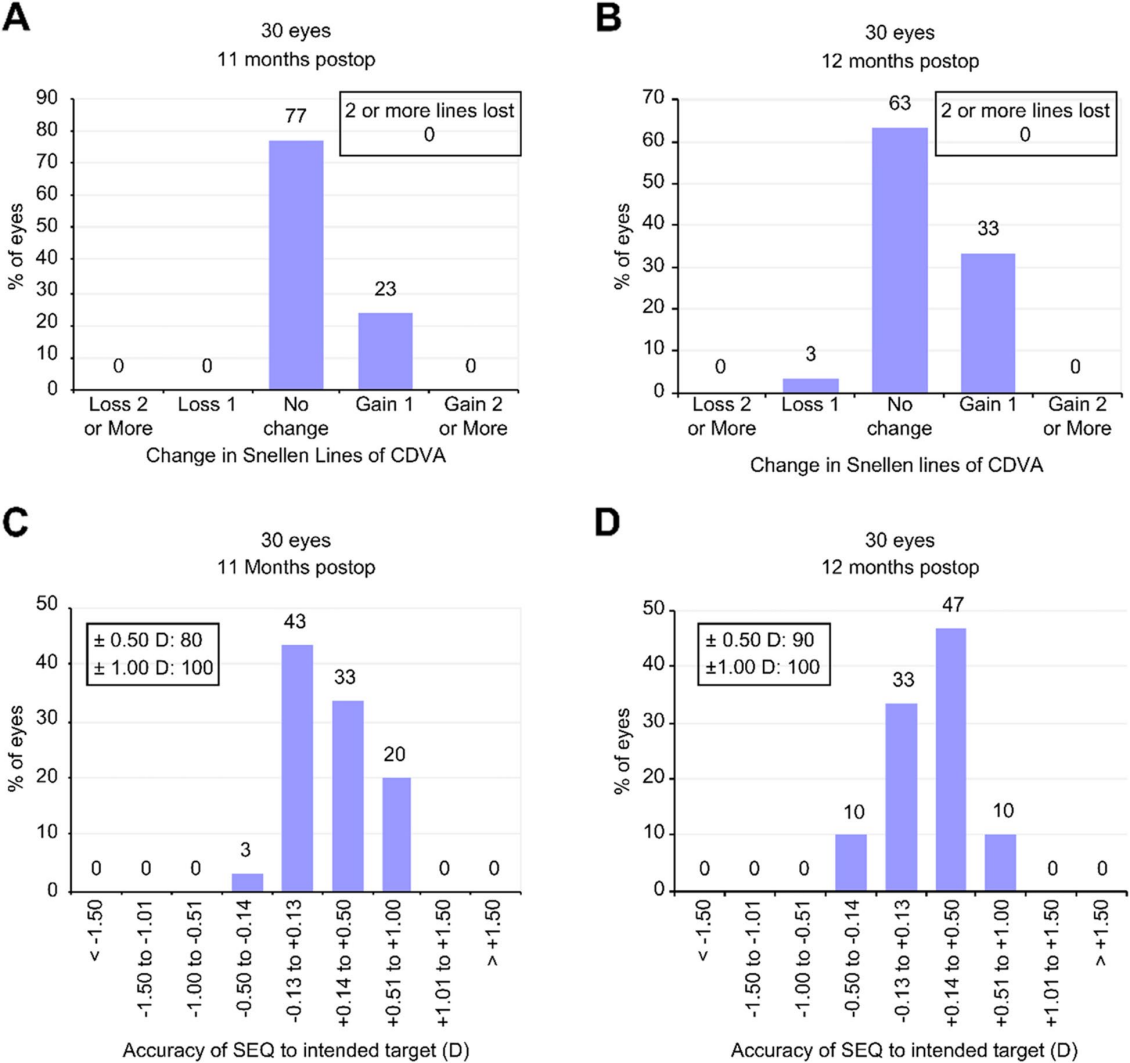
Refractive and visual outcomes, **A** and **C** = Contoura, **B** and **D** = Custom-Q (CDVA = corrected distance visual acuity, SEQ = spherical equivalent, D = diopter)

The different ablation profiles had similar safety profiles. There was no change in the CDVA in 77% of eyes subjected to Contoura or in 63% in the Custom-Q group (*p* = 0.260); furthermore, 23 and 33% of the eyes in the Contoura and Custom-Q groups, respectively, gained a line of CDVA (*p* = 0.390) (Fig. 3A and B).

#### Accuracy

There was no significant difference between the groups in the percentage of eyes that achieved ±0.50 D in the final spherical equivalent (Contoura 80% vs Custom-Q 90%; *p* = 0.278) (Fig. 3C and D).

#### Spherical equivalent attempted versus achieved

**Fig. 4:**
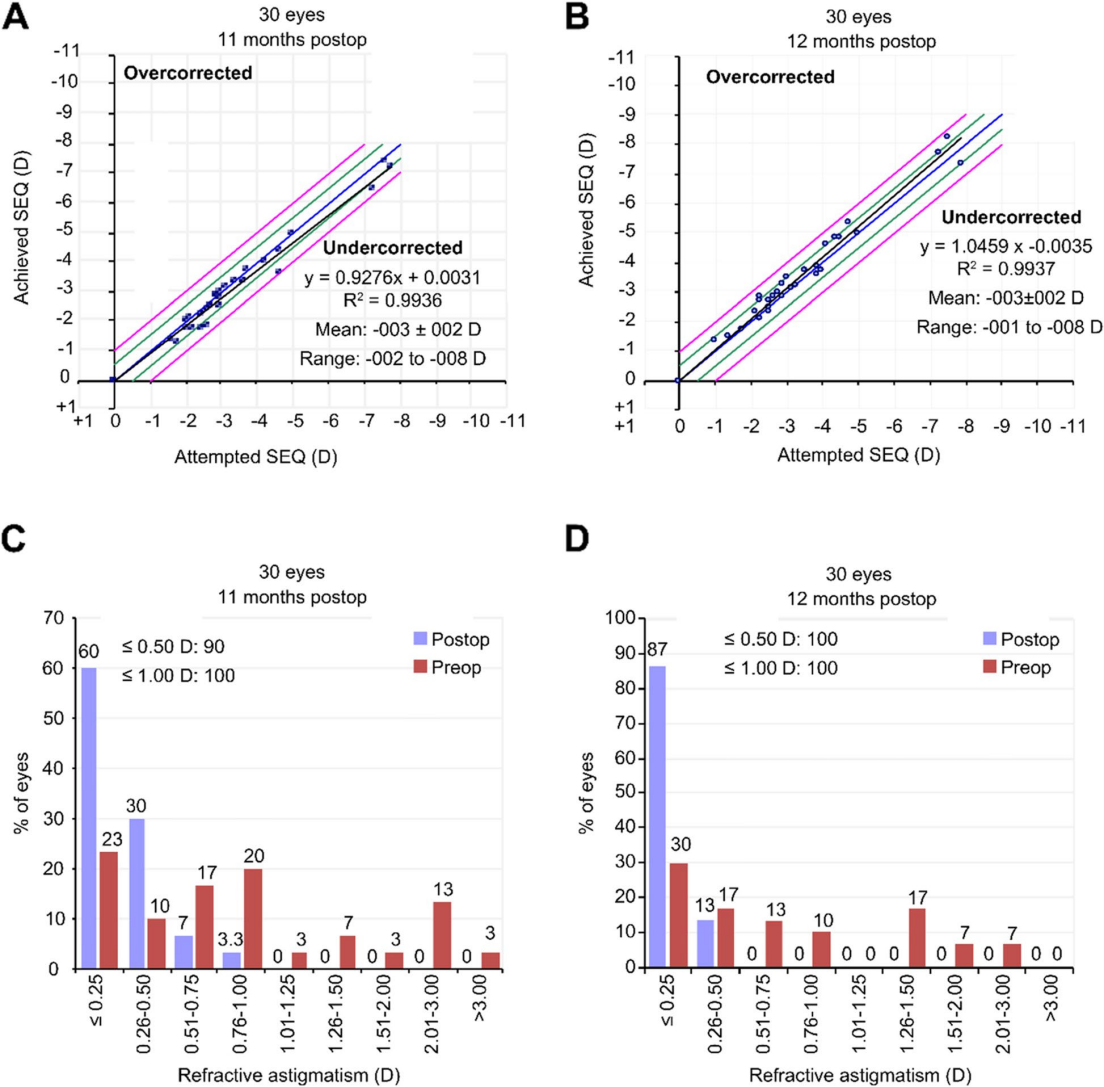
Refractive and Visual outcomes (SEQ = spherical equivalent, D = diopter)

The scatter plots show that the relationship between the attempted and achieved spherical equivalent correction in both the Contoura and Custom-Q groups at the 1-year follow-up was similar (Fig. 4A and B).

#### Astigmatism

Clinical astigmatism was corrected very effectively in both groups, with 100% of the eyes having a final result of ≤1 D. Furthermore, 90% of the eyes in the Contoura group and 100% of the eyes in the Custom-Q group achieved a residual cylinder of ≤0.50 D (*p* = 0.237) (Fig. 4C and D).

## Discussion

The main objective of our study was to detect differences in the effectiveness of two surgical techniques (Contoura and Custom-Q) in the correction of myopia with or without astigmatism. Our results indicate that at the 1-year follow-up, the two ablation profiles were shown to be effective and safe, with similar visual and refractive results in all analysis criteria and no significant disagreements between groups.

Among the parameters analysed, the UDVA is the most relevant in refractive surgery, as it reflects the patient’s primary objective: achieving good vision without glasses. In our sample, both techniques produced excellent final visual acuity: the Contoura group had a UDVA of 20/20 and 20/16 or better in 93 and 83% of eyes, while the Custom-Q group had a UDVA of 20/20 and 20/16 in 97 and 87% of eyes, respectively. In both groups, there was a significant improvement in visual acuity over 12 months.

The induction of high-order aberrations, despite being slightly higher in the Custom-Q group, was not significantly different between groups in the final analysis, contradicting the idea of the advantage of topography-guided surgery for this purpose. Regarding efficacy, safety and accuracy, it was also not possible to detect differences between the methods.

Custom-Q ablation attempts to maintain the original asphericity of the cornea, which is directly related to postoperative spherical aberration. Previous studies have shown that the Custom-Q technique causes a smaller change in the Q-value than traditional optimized ablation methods, with a consequent lower induction of spherical aberration [7, 8, 19]. Koller et al. [8] emphasized the potential of custom aspheric ablation to replace optimized ablation and demonstrated that asphericity was less affected in myopia up to − 5 D when using custom ablation than when using wavefront-optimized ablation (WFO). In a retrospective study of consecutive patients, Stojanovic et al. [19] reported good results using Custom-Q and WFO but a lower induction of spherical aberration using Custom-Q. Tawfik et al. [7] similarly suggested replacing WFO with Custom-Q. However, despite these good results and the simplicity of its programming and application, Custom-Q has not gained popularity among refractive surgeons; the good results and ease of use of WFO can be a deterrent when seeking supposedly superior methods.

Contoura topography-guided treatment, on the other hand, modifies the treatment according to the combination of two elements, the clinical and topographic cylinders. This approach has been in use for many years. In 2007, Alpins et al. [20] recommended observing the difference between cylinders to modify the treatment; Kanellopoulus [21] later developed a new surgical planning method called topography-modified refraction (TMR) based on the same principle. Furthermore, Stefano et al. [22] analysed different strategies for correcting astigmatism and found that the UDVA was better than the CDVA in 25% of eyes treated with T-CAT. Shetty et al. [23] examined 60 eyes of 30 patients and found that the UDVA was 20/20 in 97 and 93% of patients in the T-CAT group and the WFO group, respectively, with a lower induction of low- and high-order aberrations in the group. Tiwari et al. [24] evaluated 200 eyes from 100 patients and observed that the UDVA was 20/20 in 90 and 92% of patients in the WFO and T-CAT groups, respectively, with similar aberration induction in both groups. Kim et al. [25] found that the visual and refractive results were similar in contralateral eyes in the T-CAT and WFO groups; however, significantly fewer HOAs were induced with T-CAT. Ozulken et al. [26] found excellent results using T-CAT over WFO in 32 patients, with less induction of horizontal and vertical coma-type aberrations, a smaller amount of ablated tissue and 20/20 UDVA in 97% of patients. On the other hand, Zhang et al. [27] reported unfavourable results using topography-guided surgery in 432 virgin eyes of 216 patients, with less accuracy in corrected astigmatism than WFO despite demonstrating less induction of high-order aberrations. Skanchy [28] compared FDA data from three new refractive surgery platforms, namely, Wavefront-guided Visx iDesign, Wavelight TCAT (Contoura) and topography-guided Nidek EC-5000 CaTz, and found excellent results across all platforms; however, the UDVA was significantly better with T-CAT than with the other two platforms. The lack of standardization for the T-CAT is a significant limitation that prevents a comparison of results with an acceptable degree of reliability, as small differences in surgical programming between these different techniques are rarely described in detail in the literature. For this reason, we provided detailed information about the surgical schedule in the T-CAT group (Contoura) in our clinical study.

To the best of our knowledge, this is the first prospective, randomized, double-blind comparative study comparing Contoura and Custom-Q ablation treatments in normal contralateral eyes. The importance of these results is reinforced by two factors. First, because the study compares both eyes, the patients serve as their own controls, with small differences in refraction and good preoperative corrected visual acuity in both eyes. Second, treatment randomization, surgical planning, surgery, and postoperative evaluations were all performed by different investigators, which significantly increased the likelihood that any differences observed were due to the selected ablation method and not bias.

Nevertheless, this study has several limitations. First, it was not possible to assess contrast sensitivity, which could provide additional information about visual quality and perhaps reveal differences between the two techniques. Second, we did not analyse the pre and pos Q value and the main reason was that Galilei calculates asphericity through the term eccentricity (ε^2^) [29], which is different from the Q measured by Topolyzer and we thought it could cause misunderstanding. In our favor we can cite the systematic review by Zhang et al. [30] which reports no significant difference in preoperative Q-value in 11 similar studies between 2 paired groups. Third, the sample size was not sufficiently large to show the real advantages of one group over another. Furthermore, the similar results obtained for the two surgical methods may be due to their shared technical characteristics, including centring of the ablation on the apex of the cornea (instead of the pupillary axis) and attempting to maintain the initial asphericity measured by the Topolyzer Vario software. Therefore, at least in principle, the two methods may have had a similar effect on the reduced induction of spherical aberrations. These two factors (apex centring and low aspherical shift) may be more critical in determining postoperative outcomes than the correction of small HOAs in naive eyes or the slight modification of the magnitude and axis of astigmatism. However, more studies are needed to support this hypothesis.

Despite these limitations, it is important to recognize that technical advances in refractive surgery are relatively new, and several questions still need to be answered. The hypothesis of Motowanii in the Layer Yolked Reduction of Astigmatism (LYRA) protocol [31–33], in which the difference between the cylinders measured on the anterior surface of the cornea and the total astigmatism is mainly due to the interference of the HOAs, remains controversial. Other factors, such as a posterior cornea, lens, vitreous and retina, may be responsible for residual ocular astigmatism (ORA). Recent studies [34, 35] with a new analytics algorithm, the Phorcides Analytic Engine (Phorcides LLC), show promise in attempting to fill this gap. Lobanoff et al. [34] explained in his article that the FDA study for approval of Contoura only included patients demonstrating a close proximity between the clinical cylinder and that measured in the topography and excluded those who had a significant disparity between the measurements; only 24% of patients initially selected for the study were included, which significantly limited the indication of the technique by the criteria approved by the FDA.

Additionally, according to Lobanoff, in addition to anterior surface astigmatism, the Phorcides system considers corneal irregularities that contribute to high-order aberrations, posterior astigmatism and lenticular astigmatism in its ablation calculations, which may be a more effective approach than techniques guided by topography with modification of clinical refraction; however, although promising, this technology remains relatively recent, and its calculations have not yet been fully clarified. New studies are needed to write the newest chapter on customized laser surgery.

In conclusion, the correction of myopia with or without astigmatism with both the Contoura system and the Custom-Q system showed excellent visual and refractive results, but the evidence did not reveal any clear differences between these two methods after 1 year of follow-up.

## Data Availability

All data and the protocol are available from OFTALMAX, Recife-PE. Data can be accessed upon request to the authors, represented by the main author.

## Abbreviations

FemtoLASIK: Femtosecond laser-assisted in situ keratomileusis
TCAT: Topography-guided customized ablation
Custom-Q: Asphericity-guided (Q) customized ablation
UDVA: Uncorrected distance visual acuity
CDVA: Corrected distance visual acuity
VA: Visual acuity
MRSE: Manifest refractive spherical equivalent
CYL: Cylinder
PACHY: Pachymetry
PTA: Percentage of altered tissue
RMS: Total corneal aberrations root mean square
DEF: Defocus
TRE: Trefoil
SA: Spherical aberration
